# Meta-Analysis of *NUDT15* Genetic Polymorphism on Thiopurine-Induced Myelosuppression in Asian Populations

**DOI:** 10.3389/fphar.2021.784712

**Published:** 2021-12-02

**Authors:** Kanyarat Khaeso, Sariya Udayachalerm, Patcharee Komvilaisak, Su-on Chainansamit, Kunanya Suwannaying, Napat Laoaroon, Pitchayanan Kuwatjanakul, Nontaya Nakkam, Chonlaphat Sukasem, Apichaya Puangpetch, Wichittra Tassaneeyakul, Nathorn Chaiyakunapruk

**Affiliations:** ^1^ Department of Pharmacology, Faculty of Medicine, Khon Kaen University, Khon Kaen, Thailand; ^2^ Department of Pharmacotherapy, College of Pharmacy, University of Utah, Salt Lake City, UT, United States; ^3^ Department of Pediatrics, Faculty of Medicine, Khon Kaen University, Khon Kaen, Thailand; ^4^ Department of Pediatrics, Khon Kaen Hospital, Khon Kaen, Thailand; ^5^ Department of Pediatrics, Udon Thani Hospital, Udon Thani, Thailand; ^6^ Division of Pharmacogenomics and Personalized Medicine, Department of Pathology, Faculty of Medicine Ramathibodi Hospital, Mahidol University, Bangkok, Thailand; ^7^ Laboratory for Pharmacogenomics, Somdech Phra Debaratana Medical Center (SDMC), Ramathibodi Hospital, Bangkok, Thailand

**Keywords:** nucleoside diphosphate–linked moiety X-type motif 15 (NUDT15), thiopurine drugs, hematotoxicity, genetic polymorphism, precision medicine, Meta-analysis, Systematic review

## Abstract

**Backgound:** The high incidence of thiopurine-induced myelosuppression in Asians is known to be attributable to genetic variation in thiopurine metabolism. A quantitative synthesis to summarize the genetic association with thiopurine-induced myelosuppression in Asians was therefore conducted.

**Methods:** A Literature search was performed from January 2016 to May 2021 in the following databases: PubMed, Web of Science, and Embase and addition search included the studies from Zhang et al. Two reviewers independently extracted the following data: the author’s name, year of publication, ethnicity, drugs, diseases, genetic polymorphisms, onset, type of myelosuppression and results of Hardy-Weinberg equilibrium. The Newcastle-Ottawa Scale was used to assess the quality of the studies. The pooled odds ratios (OR) and 95% confidence intervals (CI) were calculated to evaluate the associations of *NUDT15* and the risk of thiopurine-induced myelosuppression stratified by onset and type of myelosuppressive. Subgroup analysis by *NUDT15* genetic polymorphisms was performed.

**Results:** A total of 30 studies was included in this meta-analysis. The overall OR for the relationship between *NUDT15* genetic polymorphisms and thiopurine-induced early onset of leukopenia and neutropenia in Asian populations were 11.43 (95% CI 7.11–18.35) and 16.35 (95% CI 10.20–26.22). Among *NUDT15* polymorphisms, NUDT15*3 showed a significantly increased risk of early leukopenia (OR 15.31; 95% CI 9.65–24.27) and early neutropenia (OR 15.85; 95% CI 8.80–28.53). A significantly higher thiopurine-induced early neutropenic risk was also found for NUDT15*2 (OR 37.51; 95% CI 1.99–708.69)*.* Whereas, NUDT15*5 and NUDT15*6 variants showed a lower risk of leukopenia.

**Conclusion:** This study suggests that NUDT15*3 and NUDT15*2 are important genetic markers of thiopurine-induced early onset of myelotoxicity in Asians, therefore, early detection of these variants before initiating thiopurine therapy is necessary.

## Introduction

Thiopurines, 6-mercaptopurine (6-MP) and azathioprine (AZA), are purine analogs that are closely related in their structures (Coulthard and Hogarth, 2005). 6-MP is widely used in the treatment of acute lymphoblastic leukemia (ALL) as part of a combination regimen at the maintenance phase and used as an immunosuppressive agent for maintaining the remission of the disease. It is also prescribed off-label for the treatment of inflammatory bowel disease (IBD) (Dean, 2012). AZA is commonly used in management of autoimmune disorders e.g. Crohn’s disease, rheumatoid arthritis, and systemic lupus erythematosus (SLE) (Zaza et al., 2010), whereas thiopurine drugs have been shown to be effective in maintaining disease remission; however, almost 30–40% of patients discontinue therapy due to adverse effects, particularly myelosuppression (Lee et al., 2015), in which its incidence is higher in Asian populations than in Caucasian populations (Kakuta et al., 2018b). Thiopurine-induced myelosuppression often causes infectious complications and some patients require therapy interruption leading to suboptimal treatment and unfavorable outcomes (Relling et al., 1999; Hindorf et al., 2006). These adverse effects are known to be caused by individual differences in thiopurine metabolism, which is affected by the genetic variations.

6-MP is metabolized into inactive 6- methyl mercaptopurine (6-MMP) by Thiopurine S-methyltransferase (TPMT) (Lennard, 1992). It is well known that *TPMT* polymorphisms result in TPMT deficiency thereby increasing concentration of 6-thioguanine nucleotide (6-TGN) levels related to myelosuppression (Weinshilboum and Sladek, 1980). The frequencies of *TPMT* polymorphisms are ethnic differences which are higher in Caucasians than in the Asian population, which may explain the high incidence of thiopurine-induced myelosuppression in Asians (Collie-Duguid et al., 1999). Recently, several lines of evidence reported that the nucleoside diphosphate–linked moiety X-type motif 15 (NUDT15) was strongly associated with thiopurine-induced myelosuppression, specifically in Asian populations (Yang et al., 2014; Yang et al., 2015; Moriyama et al., 2016). The distribution of *NUDT15* genetic polymorphism was reported to be the most common in East Asians, but rare in Caucasians (Yang et al., 2014; Yang et al., 2015; Moriyama et al., 2016). NUDT15 enzyme dephosphorylates 6-TGN then prevent the incorporation into DNA or RNA (Moriyama et al., 2016) therefore decreasing of the enzymatic activity which observed in *NUDT15* variants, particularly NUDT15*3 was leading to thiopurine-induced myelosuppression (Moriyama et al., 2016).

Although the association between the NUDT15*3 variant and thiopurine-induced myelotoxicity is well recognized, there is still controversy about the increased risk of thiopurine hematotoxicity in patients who carry other variants of this gene, particularly NUDT15*2, NUDT15*5, NUDT15*6 which exist in high allele frequencies in Asian populations (Yang et al., 2014; Yang et al., 2015; Moriyama et al., 2016; Kim et al., 2017a). A quantitative synthesis of the existing genetic association studies to summarize the magnitude of the genetic association of all common variants of *NUDT15* on thiopurine-induced toxicity was therefore conducted in order to ensure proper treatment and minimize the risk of thiopurine-induced myelosuppression in the Asian populations.

## Methods

### Data Sources and Search Strategy

A literature -search was conducted using PubMed, Web of Science, and Embase. Searches were performed using keywords and synonyms for *NUDT15* and thiopurines and relevant terms for myelosuppression. MeSH terms in PubMed were used when available. There was no language or study design restriction, but only human studies were included. In the present meta-analysis, an updated search included the studies from Zhang et al., the previously systematic review and meta-analysis (Zhang et al., 2018), and additional studies published between January 1, 2016 and May 14, 2021.

### Study Selection

Two reviewers (KK and WT) independently assessed abstracts and titles retrieved from the comprehensive searches for study inclusion. Articles from the updated search and from Zhang et al*.* were included if they met the inclusion criteria, as follows: 1) the study population was of patients treated with thiopurine drugs: 6-MP, AZA or 6-TG 2) Studied the association between genetic polymorphisms of *NUDT15* and thiopurine-induced myelosuppression in an Asian population 3) the outcomes of interest included myelosuppression (anemia, neutropenia, leukopenia, and thrombocytopenia) 4) the study provided sufficient information to calculate the genetic association with thiopurine-induced myelosuppression. Exclusion criteria are 1) studies not relevant to pharmacogenetic of *NUDT15* and *TPMT* and thiopurine-induced toxicity in Asian; 2) not clinical study; 3) review study, systematic review, meta-analysis, letters, editorials, opinion, commentaries, case report; 4) no full text available; 5) not report odds ratio or no sufficient information for calculate odd ratio or data not related to dose reduction. Any disagreements were discussed until consensus between the two reviewers could be reached.

### Data Extraction and Quality Assessment

Data extraction was performed by two independent reviewers. Any disagreement was discussed and the data checked again to arrive at an agreement. The following data were extracted from each study: the first author’s last name, year of publication, ethnicity, drugs used, disease type, genetic polymorphisms, onset and type of myelosuppression. The Hardy-Weinberg equilibrium (HWE) was tested to check if the included individuals were in equilibrium for the frequencies of genotypes (Salanti et al., 2005; Mayo, 2008). Equilibrium implies that the included individuals were likely representative of the population (Smits et al., 2005; Thakkinstian et al., 2005). The quality of the selected studies was evaluated using the Newcastle–Ottawa Scale (NOS) (Wells et al., 2014). This scale is an 8-item instrument, categorized into the following three domains: selection of participants, comparability between groups, and the assessment of exposures and outcomes. A system of stars was used to provide quality ratings for studies.

### Statistical Analysis

The pooled odd ratio (OR) and 95% CI were calculated to determine the association between genetic polymorphisms and the risk of thiopurine-induced myelosuppression. All analyses were performed with the method by DerSimonian and Laird (DerSimonian and Laird, 1986) using a random-effects model. Subgroup analysis was performed based on *NUDT15* variants. Statistical heterogeneity was assessed *via* the Q statistic and I^2^ tests (Higgins and Thompson, 2002). *p* ≤ 0.10 indicated heterogeneity between studies. I^2^ values of 25 and 50% denoted low heterogeneity and moderate heterogeneity, across studies (Higgins et al., 2003). The Funnel plot, Begg test, and Egger test were used to evaluate small study effect (Begg and Berlin, 1989). All analyses were performed in STATA version 13.0 (StataCorp, College Station, Texas, USA).

## Results

### Study Selection

**FIGURE 1:**
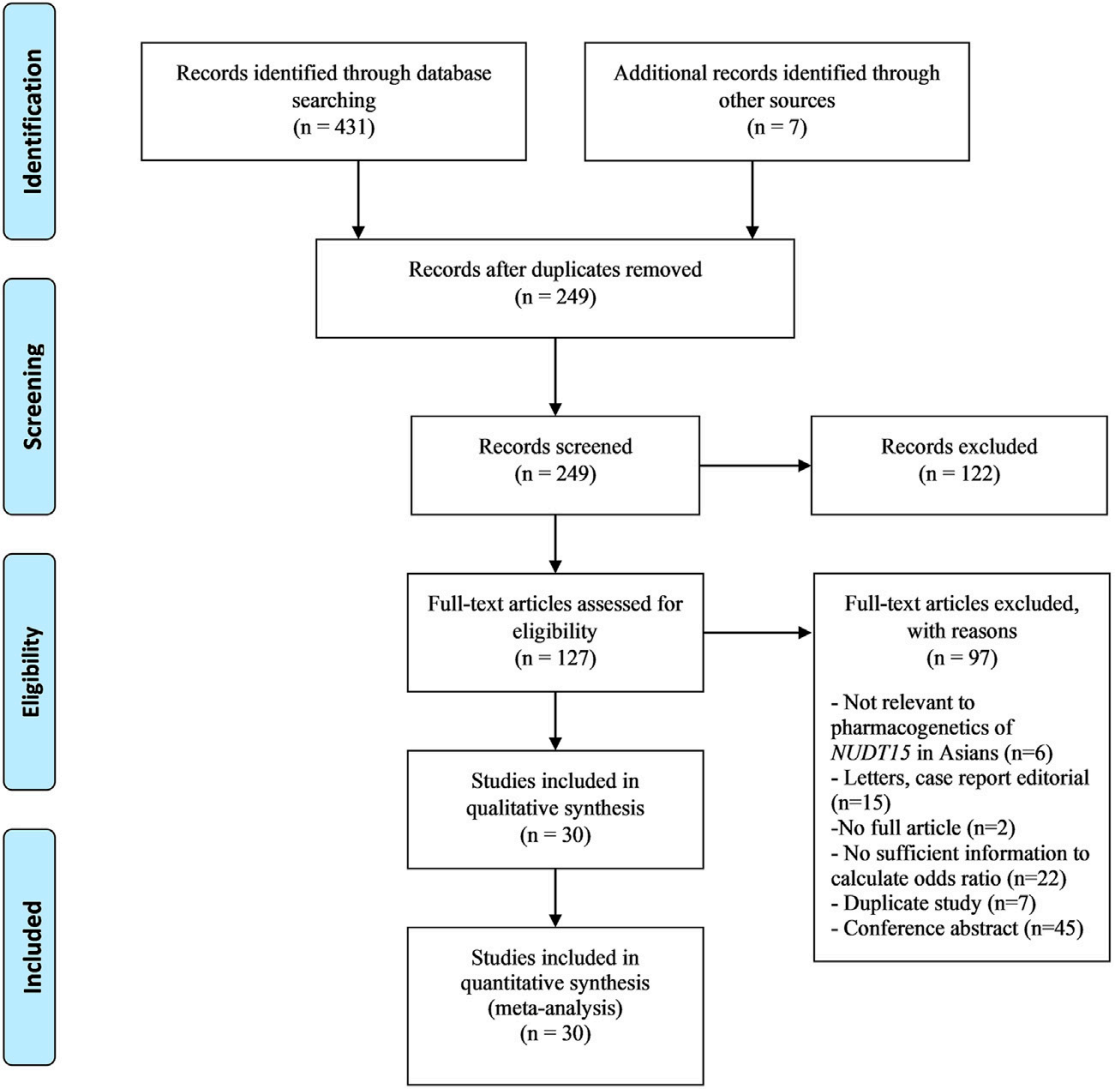
Study selection process.

A total of 431 studies were identified from an updated literature search while an additional seven studies from a previous systematic meta-analysis by Zhang et al. (2018) were reviewed. Of the original 431 articles, 30 studies were included in the meta-analysis. No additional articles were identified via a review of the bibliographies of the included studies. (Figure 1).

### Study Characteristics

Characteristics of 30 studies are described in Table 1. All studies were conducted among Asians populations. There were 1,167 cases with leukopenia and 240 cases with neutropenia. Among these, 14 studies were conducted in IBD (Yang et al., 2014; Asada et al., 2016; Kakuta et al., 2016; Zhu et al., 2016; Chao et al., 2017; Sato et al., 2017; Shah et al., 2017; Kakuta et al., 2018a; Sutiman et al., 2018; Wang et al., 2018; Akiyama et al., 2019; Banerjee et al., 2020; Kang et al., 2020; Xu et al., 2020), nine studies in ALL (Tanaka et al., 2015; Chiengthong et al., 2016; Tanaka et al., 2018; Zhou et al., 2018; Buaboonnam et al., 2019; Choi et al., 2019; Kodidela et al., 2020; Puangpetch et al., 2020; Ramalingam et al., 2021) and seven studies in other autoimmune diseases (autoimmune hepatitis, dermatological diseases and rheumatological diseases) (Kim et al., 2017b; Fei et al., 2018; Yang et al., 2019a; Fan et al., 2019; Huang et al., 2020; Su et al., 2020; Miao et al., 2021).

**TABLE 1:** Characteristics of all analyses.

Source	Nationality	Disease type	Sample size	Drug	SNP	Toxic criteria	Onset	Myelosup pression type	No. positive for genetic variation/Total no.	
									Case	Control
Yang et al. (2014)	Korean	Crohn’s disease	978	AZA	NUDT15*3	WBC <3,000 cells/mm^3^	within 8 weeks	Early leukopenia	59/66	131/912
						WBC <3,000 cells/mm^3^	after the first 8 weeks	Late leukopenia	88/280	102/698
Tanaka et al. (2015)	Japanese	ALL	92	6-MP	NUDT15*3	WBC <2 × 10^9^/L	within first 60 days	Early leukopenia	10/22	14/70
Asada et al. (2016)	Japanese	IBD	161	AZA/6-MP	NUDT15*3	WBC <3,000/uL	within 8 weeks	Early leukopenia	4/6	30/115
						WBC <3,000/uL	after the first 8 weeks	Late leukopenia	16/39	14/116
Chiengthong et al. (2016)	Thai	ALL	82	6-MP	NUDT15*3	ANC <500 cells/µL	at month 2	Early neutropenia	3/6	9/76
					NUDT15*3	ANC <500 cells/µL	at month 4	Late neutropenia	9/21	3/61
Kakuta et al. (2016)	Japanese	IBD	135	AZA/6-MP	NUDT15*3	WBC <3,000 mm^−3^	within 8 weeks	Early leukopenia	9/10	19/125
						WBC <3,000 mm^−3^	after 8 weeks	Late leukopenia	6/24	12/87
Zhu et al. (2016)	Han Chinese	CD	253	AZA/6-MP	NUDT15*3	WBC <3.5 × 10^9^/L	0–8 weeks	Early leukopenia	19/27	38/226
						WBC <3.5 × 10^9^/L	8–24 weeks	Late leukopenia	16/22	41/231
Chao et al. (2017)	Han Chinese	IBD	732	AZA/6-MP	NUDT15*3	WBC <3,500 mm^−3^	0–8 weeks	Early leukopenia	43/70	132/662
						WBC <3,500 mm^−3^	8–24 weeks	Late leukopenia	23/43	152/689
					NUDT15*6	WBC <3,500 mm^−3^	0–8 weeks	Early leukopenia	18/70	66/662
						WBC <3,500 mm^−3^	8–24 weeks	Late leukopenia	10/43	74/689
					NUDT15*5	WBC <3,500 mm^−3^	0–8 weeks	Early leukopenia	6/70	11/662
						WBC <3,500 mm^−3^	8–24 weeks	Late leukopenia	2/43	15/689
Kim et al. (2017b)	Korean	Neuro immunological diseases	92	AZA	NUDT15*3	WBC <3,500 cells/μl	within 8 weeks	Early leukopenia	6/7	7/77
						WBC <3,500 cells/μl	after 8 weeks	Late leukopenia	2/13	11/71
Sato et al. (2017)	Japanese	CD, UC, IBD unclassified, or intestinal Behçet disease	160	AZA/6-MP	NUDT15*3	WBC <3,000/μL	within 8 weeks	Early leukopenia	13/16	27/133
						WBC <3,000/μL	after 8 weeks	Late leukopenia	17/43	23/106
					NUDT15*6	WBC <3,000/μL	within 8 weeks	Early leukopenia	2/16	8/133
						WBC <3,000/μL	after 8 weeks	Late leukopenia	5/43	5/106
					NUDT15*5	WBC <3,000/μL	within 8 weeks	Early leukopenia	0/16	2/133
						WBC <3,000/μL	after 8 weeks	Late leukopenia	2/43	0/106
Shah et al. (2017)	Indian	UC, CD, AIH	69	AZA/6-MP	NUDT15*3	WBC <3,000/mm^3^	within 8 weeks	Early leukopenia	4/4	5/65
						WBC <3,000/mm^3^	after 8 weeks	Late leukopenia	2/2	7/67
Fei et al. (2018)	Chinese	Auto-immune diseases	87	AZA	NUDT15*3	WBC <3.5 × 10^9^/L	before 8 weeks	Early leukopenia	17/21	11/66
						WBC <3.5 × 10^9^/L	after 8 weeks	Late leukopenia	1/2	27/86
Kakuta et al. (2018a)	Japanese	CD, UC, or BD	2,627	AZA/6-MP	NUDT15*3	WBC <3,000/lL	<8 weeks	Early leukopenia	66/80	258/1,202
Sutiman et al. (2018)	Asians (Chinese, Indian, Malay, others)	IBD	129	AZA/6-MP	NUDT15*3	WBC <3 × 10^9^/L	NA	Leukopenia	7/10	11/119
						ANC <1.5 × 109/L	NA	Neutropenia	6/10	12/119
					NUDT15*6	WBC <3 × 10^9^/L	NA	Leukopenia	3/10	8/119
						ANC <1.5 × 10^9^/L	NA	Neutropenia	2/10	9/119
Tanaka et al. (2018)	Japanese	ALL	95	6-MP	NUDT15*3	WBC <2.0 × 10^9^/L	NA	Leukopenia	19/38	6/57
					NUDT15*5	WBC <2.0 × 10^9^/L	NA	Leukopenia	4/38	1/57
Wang et al. (2018)	Chinese	IBD	219	AZA	NUDT15*3	WBC <3.5 × 10^9^/L	NA	Leukopenia	8/19	8/61
Zhou et al. (2018)	Chinese	ALL	105	6-MP	NUDT15*3	WBC <2 × 10^9^/L	during the first 60 days	Early leukopenia	11/15	20/90
Akiyama et al. (2019)	Japanese	IBD	83	AZA/6-MP	NUDT15*3	WBC <3,000/mm^3^	NA	Leukopenia	11/18	10/63
Buaboonnam et al. (2019)	Thai	ALL	102	6-MP	NUDT15*3	ANC <500/mm^3^	at 3 months	Late neutropenia	12/18	12/84
Choi et al. (2019)	Korean	ALL	139	6-MP	NUDT15*3	WBC <1.5 × 109 L^−1^	NA	Leukopenia	5/7	25/132
						ANC <0.5 × 109 L^−1^	NA	Neutropenia	3/4	27/132
Fan et al. (2019)	Chinese	AIH	149	AZA	NUDT15*3	WBC <3 × 10^9^/L	during first 8 weeks	Early leukopenia	11/12	15/137
Yang et al. (2019a)	Han Chinese	Auto-immune diseases	86	AZA	NUDT15*3	WBC <3.5 × 10^9^/L	NA	Leukopenia	11/19	7/67
					NUDT15*5	WBC <3.5 × 10^9^/L	NA	Leukopenia	0/19	3/67
					NUDT15*6	WBC <3.5 × 10^9^/L	NA	Leukopenia	3/19	2/67
Huang et al. (2020)	Han Chinese	Dermatological diseases	56	AZA	NUDT15*3	ANC <1,500/mm^3^	within 8 weeks	Early neutropenia	5/7	15/49
						ANC <1,500/mm^3^	after 8 weeks	Late neutropenia	3/5	17/51
Kang et al. (2020)	Korean	IBD	167	AZA	NUDT15*3	WBC <3,000/μL	NA	Leukopenia	13/32	14/114
					NUDT15*5	WBC <3,000/μL	NA	Leukopenia	2/21	3/103
					NUDT15*6	WBC <3,000/μL	NA	Leukopenia	1/20	0/100
Kodidela et al. (2020)	South Indian	ALL	73	6-MP	NUDT15*3	grade 3–4	within the first 100 days	Early leukopenia	12/39	2/32
						hematological toxicities				
Puangpetch et al. (2020)	Thai	ALL	100	6-MP	NUDT15*2	ANC <500/mm^3^	weeks 1–8	Early neutropenia	5/24	0/66
						ANC <500/mm^3^	weeks 9–24	Late neutropenia	2/41	3/49
					NUDT15*3	ANC <500/mm^3^	weeks 1–8	Early neutropenia	3/22	1/67
						ANC <500/mm^3^	weeks 9–24	Late neutropenia	0/39	4/50
					NUDT15*6	ANC <500/mm^3^	weeks 1–8	Early neutropenia	4/23	2/68
						ANC <500/mm^3^	weeks 9–24	Late neutropenia	6/45	0/46
Banerjee et al. (2020)	Indian	IBD	935	AZA	NUDT15*3	WBC <3 × 10^9^/L	NA	Leukopenia	54/81	80/854
						ANC <1.5 × 10^9^/L	NA	Neutropenia	49/70	85/865
Su et al. (2020)	Chinese	Rheumatological disease	70	AZA	NUDT15*3	WBC <3.5 × 10^9^/L	NA	Leukopenia	13/28	6/42
Xu et al. (2020)	Chinese	IBD	159	AZA	NUDT15*3	WBC <3.5 × 10^9^/L	NA	Leukopenia	14/37	19/122
Miao et al. (2021)	Chinese	AIH	113	AZA	NUDT15*3	WBC <4 × 10^9/^L	NA	Leukopenia	9/15	16/98
						ANC <2 × 10^9^/L	NA	Neutropenia	7/10	18/103
Ramalingam et al. (2021)	South Indian	ALL	127	6-MP	NUDT15*3	ANC <2000 cells/mm^3^	NA	Neutropenia	7/28	5/99

Note: WBC, white blood cell; ANC, absolute neutrophil count; NA, not available. SNP, it is not a value. It is the name of gene and according to the nomenclature of genes, italic text should be used.

Myelosuppression was categorized based on the onset and characteristics of blood cell type, including of leukopenia and neutropenia. The onset of myelosuppression within 8 weeks was defined as early whereas the onset after 8 weeks was defined as late. For those studies without a clear description of onset of myelosuppression, the onset was classified based on duration of the study. Among studies conducted in ALL patients, the 6-MP dose was 40–75 mg/m^2^/d, while the studies conducted in IBD or autoimmune diseases was AZA 0.5–3 mg/kg/d. The distribution of observed alleles and expected alleles of each genetic variation were consistent with HWE, except for three studies (Tanaka et al., 2015; Kakuta et al., 2018a; Tanaka et al., 2018).

### Quality Assessment

The methodological quality of all studies is summarized as a mean Newcastle-Ottawa Scale score of 8 (range, 7–9; maximum score, 9, see Supplementary Table S2).

## Quantitative Synthesis

Association between genetic polymorphisms involved in thiopurine metabolism and risk of myelosuppression.

### NUDT15*3 Polymorphisms (rs116855232)

**FIGURE 2:**
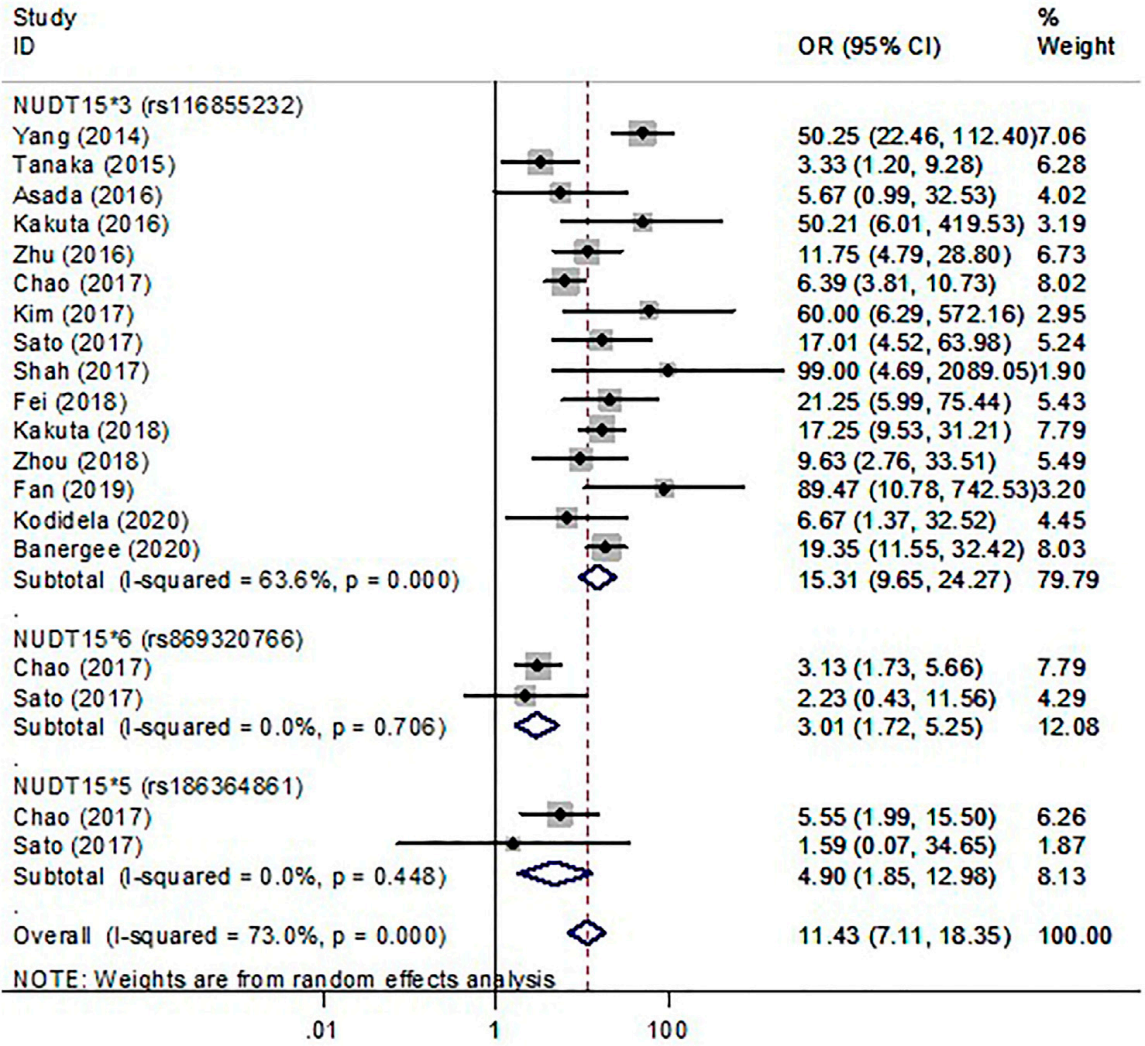
The forest plots for the association of *NUDT15* variants with thiopurine-induced early leukopenia. *Note: Width of the box indicates the precision of the estimates; diamond, the overall summary estimate for the analysis (width of the diamond represents the 95% CI)*.

**FIGURE 3:**
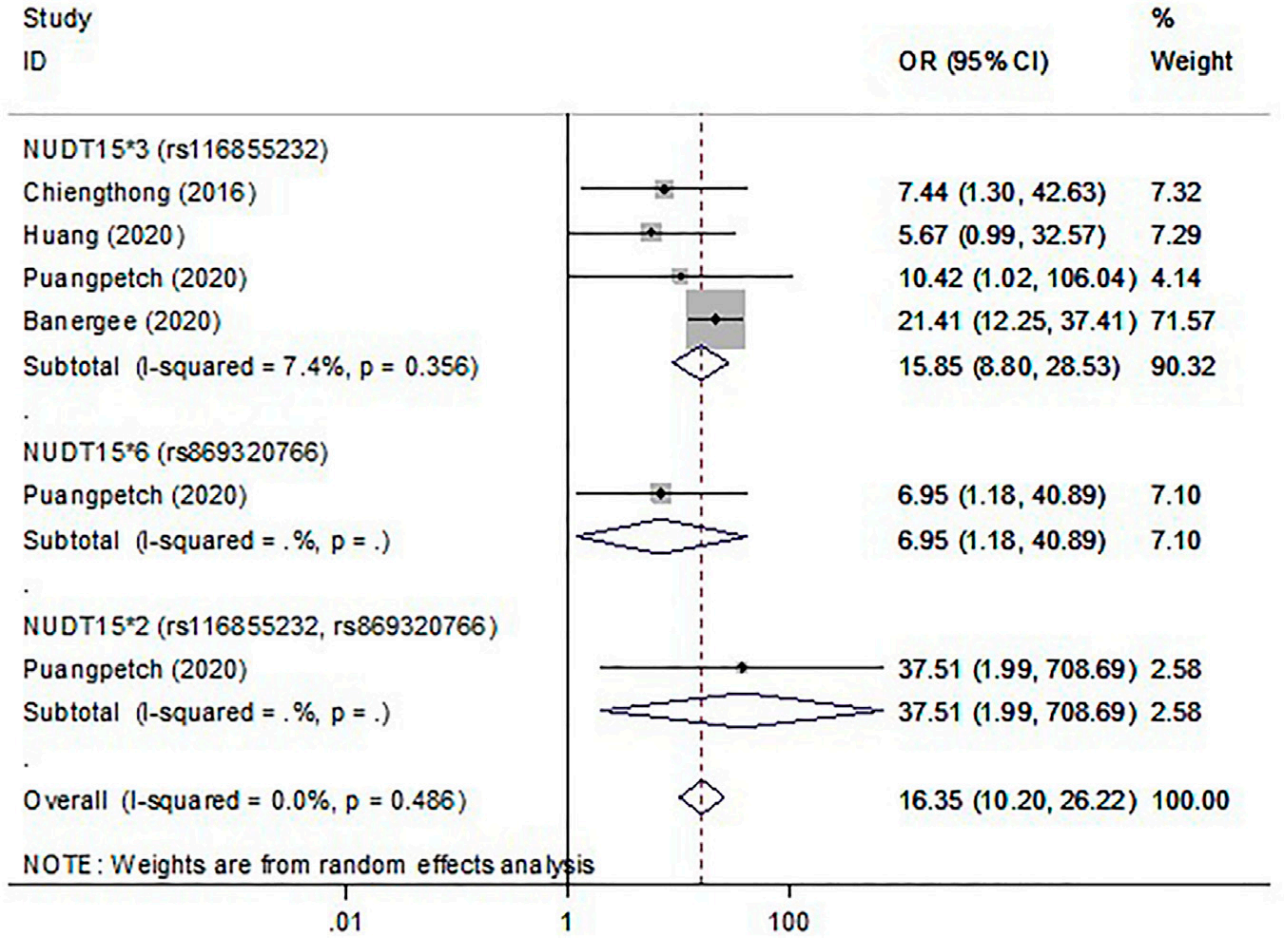
The forest plots for the association of *NUDT15* variants with thiopurine-induced early neutropenia. *Note: Width of the box indicates the precision of the estimates; diamond, the overall summary estimate for the analysis (width of the diamond represents the 95% CI)*.

In thirty studies with NUDT15*3, there were 476 cases with early leukopenia from 15 studies, 691 cases with late leukopenia from 19 studies. Of these, 338 cases (71%) with early onset and 281 cases (40.67%) with late onset carried the NUDT15*3 variant. The higher risk for development of early leukopenia was found to be significantly associated with NUDT15*3 carriers (OR 15.31; 95% CI 9.65–24.27, I^2^ = 63.6%) (Figure 2) compared to those patients with late onset (OR 4.9; 95% CI 3.56–6.74, I^2^ = 47.4) (Supplementary Figure S1). In addition, there were 105 cases with early neutropenia. Of these, 60 cases (57.14%) carried NUDT15*3. The study also found a strong relationship between NUDT15*3 and risk of early neutropenia in studied patients (OR 15.85; 95% CI 8.8–28.53, I^2^ 7.4%, *p* = 0.356) (Figure 3).

### NUDT15*5 (rs186364861), NUDT15*6 (rs869320766) and NUDT15*2 (rs116855232, rs869320766)

Six studies were included to determine the association of NUDT15*5 and NUDT15*6 with the risk of myelosuppression. Overall, with those who carried either NUDT15*5 or NUDT15*6, 15.12% (26 of 172) cases had early leukopenia whereas 10.70% (32 of 299) cases had late leukopenia. For neutropenia, 17.39% (4 of 23) cases with early onset and 14.55% (8 of 55) cases with late onset carried NUDT15*6. The OR for early and late leukopenia was about 3.01–4.9 for NUDT15*5 and NUDT15*6 variants (Figure 2, Supplementary Figure S1). Only one study showed a significant 6.95-fold higher risk for NUDT15*6 variant carriers to develop early neutropenia (OR 6.95; 95% CI 1.18–40.89) (Figure 3). There was one study reporting the significant association between NUDT15*2 and early neutropenia in ALL patients (OR 37.51; 95% CI 1.99–708.69) (Figure 3) but not for late neutropenia (Supplementary Figure S2).

### Small Study Effect

Begg’s and Egger’s test were performed to assess for small study effect. The Egger’s test and Begg’s test were not significant for all analysis (*p* > 0.05, see Supplementary Table S1), except for late neutropenia. However, no asymmetry was found in the funnel plot indicating a lack of evidence for a small study effect (see Supplementary Figures S3–6).

## Discussion

Recently, a number of studies suggested that NUDT15*3 was a novel predictor of thiopurine-induced myelosuppression in Asians (Yang et al., 2014; Yang et al., 2015; Moriyama et al., 2016). To date, more than 20 variants of the *NUDT15* gene have been reported (Yang et al., 2019b). The common variant alleles are NUDT15*2 (rs869320766; c.36_37insGGAGTC and rs116855232; c.415C > T), NUDT15*3 (rs116855232; c.415C > T), NUDT15*5 (rs186364861; c.52G > A), and NUDT15*6 (rs869320766; c.36_37insGGAGTC) (Moriyama et al., 2016) in which NUDT13*3 is the most prevalent in Asian populations (Yang et al., 2014; Yang et al., 2015; Moriyama et al., 2016; Kim et al., 2017a; Khaeso et al., 2021). This current study’s results indicate that the overall OR for the relationship between *NUDT15* genetic polymorphisms and thiopurine-induced early onset of leukopenia and neutropenia were 11.43 (95%CI 7.11–18.35) and 16.35 (95% CI 10.20–26.22). The higher risk was noted in patients who carried NUDT15*3 more than any other variant with an OR of 15.31 for early leukopenia and 15.85 for early neutropenia. In addition, an almost 38-fold increase of risk of early neutropenia was also found for NUDT15*2 carrier patients.

NUDT15*3 and NUDT15*2 showed a 100*%* loss of enzyme activity (Moriyama et al., 2016). The recent Clinical Implementation Consortium (CPIC) Guidelines for Thiopurine classified an individual carrying one normal function allele with NUDT15*2 or NUDT15*3 allele as an intermediate metabolizer whereas an individual carrying these two no function alleles were poor metabolizers (Relling and Schwab, 2019). It has been reported that patients with NUDT15*1/*2 which contain both rs869320766 and rs116855232 had a similar degree of 6-MP intolerance as the NUDT15*1/*3 (which contained a single rs116855232 SNP) (Moriyama et al., 2016)*.* A similar result was reported in that the NUDT15*2 variant showed an approximate 38-fold higher risk of early neutropenia in ALL patients who were treated with 6-MP (Puangpetch et al., 2020). This suggested that these two variants of NUDT15 proteins may have exhibited similar enzymatic activity (Moriyama et al., 2016).

Unlike NUDT15*3 and NUDT15*2, an *in vitro* study reported that NUDT15*5 and NUDT15*6 showed a loss of enzyme activity of about 50–60%, however, *in vivo* activities of these enzymes were not quite clear (Moriyama et al., 2016). Previous studies showed controversy about the increased risk of thiopurine hematotoxicity in patients who carried these variant alleles, particularly NUDT15*6 (Sato et al., 2017; Sutiman et al., 2018; Tanaka et al., 2018). The previous meta-analysis has reported a lower diagnostic accuracy for NUDT15*6 and NUDT15*5 compared to NUDT15*3 (Cargnin et al., 2018), consistent with the current results that revealed a lower risk of OR in patients who carried NUDT15*5 or NUDT15*6 with the risk of myelosuppression compared to NUDT15*3.

The results from this meta-analysis showed that the lower number of OR was found in late onset of myelosuppression and that these may be because thiopurine toxicity often occurs in the first few months of the maintenance phase. The reason may be due to the fact that practically, the thiopurine dose was gradually adjusted to the tolerated dose which resulted in no myelosuppressive effect. Therefore NUDT15*3 polymorphism had the increased risk of thiopurine-induced leukopenia and neutropenia in particular, as early as the first 2 months of the maintenance phase of treatment.

One limitation that deserves discussion is a significant heterogeneity observed amongst studies evaluating the association between with NUDT15*3 carriers and early leukopenia. We could not explore the cause of this heterogeneity. Despite the fact that we used a random-effects model to pool the results across studies, the results of the meta-analysis for early leukopenia should be interpreted with caution.

## Conclusion

In summary, this study found a strong relationship between NUDT15*3 and NUDT15*2 variants and thiopurine-induced early onset myelosuppression. The rs116855232 SNP which exists in both of these variants appears to be a key SNP for thiopurine-induced hematoxtoxicity. The genotyping of the rs116855232 which has a high prevalence in Asian populations should be considered prior prescribing thiopurine drugs in order to predict the risk of early myelosuppression.

## Data Availability

The original contributions presented in the study are included in the article/Supplementary Material, further inquiries can be directed to the corresponding authors.
