# Supplementary material for: Meta-Analysis of NUDT15 Genetic Polymorphism on Thiopurine-Induced Myelosuppression in Asian Populations
Source: Front Pharmacol. 2021 Dec 2;12:784712. doi: 10.3389/fphar.2021.784712 (PMC8675242; doi:10.3389/fphar.2021.784712)
Supplement: Supplementary file 1 [file DataSheet1.docx]

Supplementary Material

**Supplementary Figure1.** The forest plots for the association of *NUDT15* variants with thiopurine- induced late leukopenia

*Note: Forest plot of subgroup analyses according to NUDT15 polymorphisms. Width of the box indicates the precision of the estimates; the diamond, the overall summary estimate for the analysis (width of the diamond represents the 95% CI).*

**Supplementary Figure2.** The forest plots for the association of *NUDT15* variants with thiopurine- induced late neutropenia

*Note: A Forest plot of subgroup analyses according to NUDT15 polymorphisms. Width of the box indicates the precision of the estimates; the diamond, the overall summary estimate for the analysis (width of the diamond represents the 95% CI).*


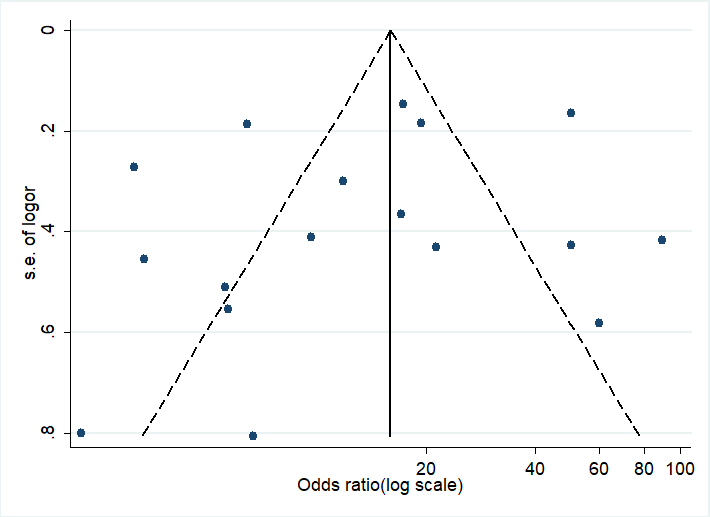


**Supplementary Figure3.** Funnel plots for the assessment of publication bias of thiopurine- induced early leukopenia


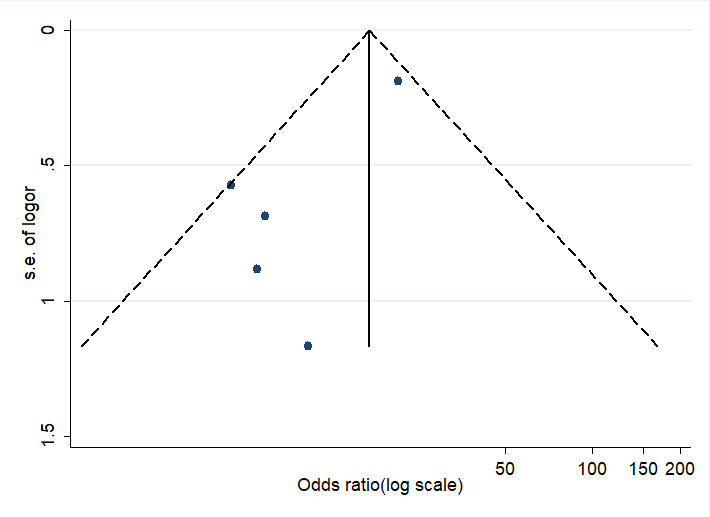


**Supplementary Figure4.** Funnel plots for the assessment of publication bias of thiopurine- induced early neutropenia


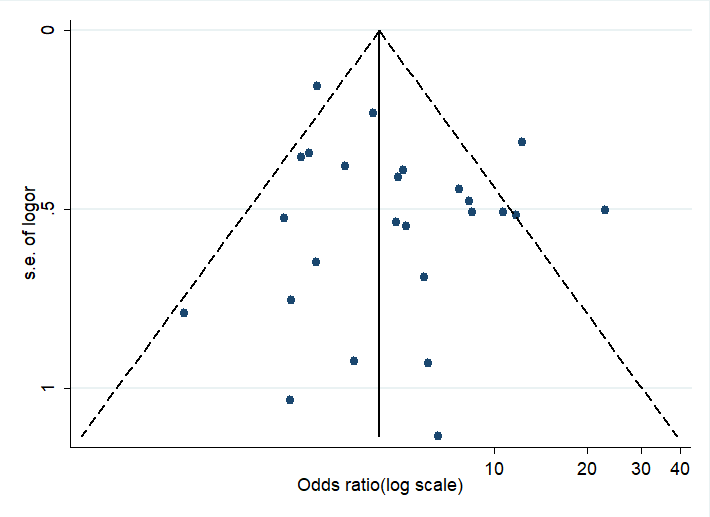


**Supplementary Figure5.** Funnel plots for the assessment of publication bias of thiopurine- induced late leukopenia


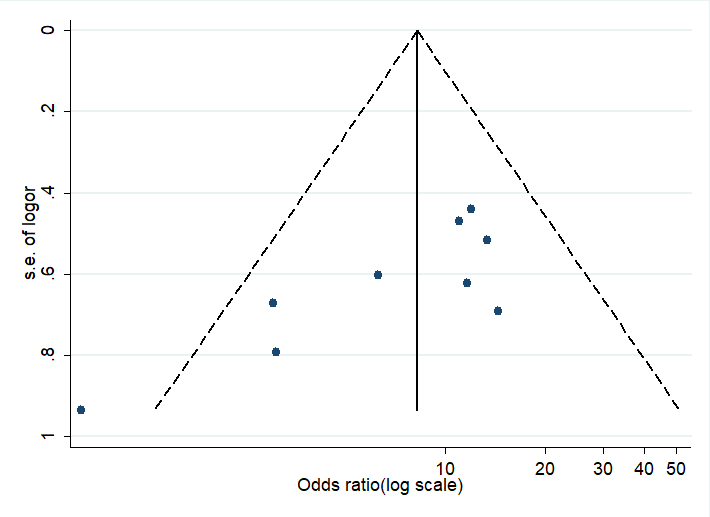


**Supplementary Figure6.** Funnel plots for the assessment of publication bias of thiopurine- induced late neutropenia

**Supplementary Table1.** Publication bias was investigated by Egger’s and Begg’s test (P<0.05 was considered to indicate statistical significance)

| **Myelosuppression** | **Egger’s test** | **Begg’s test** |
| --- | --- | --- |
| Early leukopenia | 0.409 | 0.484 |
| Late leukopenia | 0.190 | 0.981 |
| Early neutropenia | 0.062 | 0.806 |
| Late neutropenia | 0.014 | 0.048 |

**Supplementary Table2. The Newcastle-Ottawa quality assessment scale**

| **Study** | **Selection** | **Comparability** | **Outcome** | **Total score** |
| --- | --- | --- | --- | --- |
| Yang et al. 2014 (Yang et al., 2014) | 4 | 2 | 3 | 9 |
| Tanaka et al. 2015 (Tanaka et al., 2015) | 4 | 2 | 3 | 9 |
| Asada et al. 2016 (Asada et al., 2016) | 4 | 2 | 3 | 9 |
| Chiengthong et al. 2016 (Chiengthong et al., 2016) | 4 | 1 | 3 | 8 |
| Kakuta et al. 2016 (Kakuta et al., 2016) | 4 | 1 | 3 | 8 |
| Zhu et al. 2016 (Zhu et al., 2016) | 4 | 1 | 3 | 8 |
| Chao et al. 2017 (Chao et al., 2017) | 4 | 2 | 3 | 9 |
| Kim et al. 2017 (Kim et al., 2017b) | 3 | 1 | 3 | 7 |
| Sato et al. 2017 (Sato et al., 2017) | 4 | 1 | 3 | 8 |
| Shah et al. 2017 (Shah et al., 2017) | 4 | 1 | 3 | 8 |
| Fei et al. 2018 (Fei et al., 2018) | 3 | 1 | 3 | 7 |
| Kukuta et al. 2018 (Kakuta et al., 2018a) | 4 | 1 | 3 | 8 |
| Sutiman et al. 2018 (Sutiman et al., 2018) | 4 | 1 | 3 | 8 |
| Tanaka et al. 2018 (Tanaka et al., 2018) | 4 | 1 | 3 | 8 |
| Wang et al. 2018 (Wang et al., 2018) | 4 | 1 | 3 | 8 |
| Zhou et al. 2018 (Zhou et al., 2018) | 4 | 1 | 3 | 8 |
| Akiyama et al. 2018 (Akiyama et al., 2019) | 4 | 1 | 3 | 8 |
| Buaboonnam et al. 2019 (Buaboonnam et al., 2019) | 4 | 1 | 3 | 8 |
| Choi et al. 2019 (Choi et al., 2019) | 4 | 1 | 3 | 8 |
| Fan et al. 2019 (Fan et al., 2019) | 4 | 1 | 3 | 8 |
| Yang et al. 2019 (Yang et al., 2019a) | 3 | 1 | 3 | 7 |
| Huang et al. 2020 (Huang et al., 2020) | 3 | 1 | 3 | 7 |
| Kang et al. 2020 (Kang and Kim, 2020) | 4 | 1 | 3 | 8 |
| Kodidela et al. 2020 (Kodidela et al., 2020) | 4 | 1 | 3 | 8 |
| Puangpetch et al. 2020 (Puangpetch et al., 2020) | 4 | 2 | 3 | 9 |
| Banergee et al. 2020 (Banerjee et al., 2020) | 4 | 2 | 3 | 9 |
| Song-Sen Su et al. 2020 (Su et al., 2020) | 3 | 1 | 3 | 7 |
| Ye Xu et al. 2020 (Xu et al., 2020) | 4 | 2 | 3 | 9 |
| Miao et al. 2021 (Miao et al., 2021) | 4 | 1 | 3 | 8 |
| Ramalingam et al. 2021 (Ramalingam et al., 2021) | 4 | 2 | 3 | 9 |

*Note: The quality of the selected studies was evaluated using the Newcastle–Ottawa Scale (NOS) (Wells et al.). This scale is an 8-item instrument, categorized into the following 3 domains: selection of participants (maximum score of 4), comparability between groups (maximum score of 2), and the assessment of exposures and outcomes (maximum score of 3).*
